# Evolutionary Constraints on the Norovirus Pandemic Variant GII.4_2006b over the Five-Year Persistence in Japan

**DOI:** 10.3389/fmicb.2017.00410

**Published:** 2017-03-13

**Authors:** Hironori Sato, Masaru Yokoyama, Hiromi Nakamura, Tomoichiro Oka, Kazuhiko Katayama, Naokazu Takeda, Mamoru Noda, Tomoyuki Tanaka, Kazushi Motomura

**Affiliations:** ^1^Pathogen Genomics Center, National Institute of Infectious DiseasesTokyo, Japan; ^2^Department of Virology II, National Institute of Infectious DiseasesTokyo, Japan; ^3^Graduate School of Infection Control Sciences, Kitasato UniversityTokyo, Japan; ^4^Research Institute for Microbial Diseases, Osaka UniversityOsaka, Japan; ^5^Thailand-Japan Research Collaboration Center on Emerging and Re-emerging InfectionsNonthaburi, Thailand; ^6^National Institute of Health SciencesTokyo, Japan; ^7^Sakai City Institute of Public HealthOsaka, Japan

**Keywords:** norovirus, pandemic strain, GII.4, capsid, molecular evolution, evolutionary constraints

## Abstract

Norovirus GII.4 is a major cause of global outbreaks of viral gastroenteritis in humans, and has evolved by antigenic changes under the constantly changing human herd immunity. Major shift in the pandemic GII.4 strain periodically occurs concomitant with changes in the antigenic capsid protein VP1. However, how the newly emerged strain evolves after the onset of pandemic remains unclear. To address this issue, we examined molecular evolution of a pandemic lineage, termed the GII.4_2006b, by using the full-length viral genome and VP1 sequences (*n* = 317) from stools collected at 20 sites in Japan between 2006 and 2011. Phylogenetic tree showed a radial diversification of the genome sequences of GII.4_2006b, suggesting a rapid genetic diversification of the GII.4_2006b population from a few ancestral variants. Impressively, amino acid sequences of the variable VP1 in given seasons remained as homogeneous as those of viral enzymes under annual increase in the nucleotide diversity in the VP1 coding region. The Hamming distances between the earliest and subsequent variants indicate strong constraints on amino acid changes even for the highly variable P2 subdomain. These results show the presence of evolutionary constraints on the VP1 protein and viral enzymes, and suggest that these proteins gain near maximal levels of fitness benefits in humans around the onset of the outbreaks. These findings have implications for our understanding of molecular evolution, mechanisms of the periodic shifts in the pandemic NoV GII.4 strains, and control of the NoV GII.4 pandemic strain.

## Introduction

Norovirus (NoV) is a non-enveloped RNA virus that belongs to the family *Caliciviridae*. NoV is divided into multiple genogroups, which are further subgrouped into more than 40 genotypes (Zheng et al., 2006; Donaldson et al., 2010; Robilotti et al., 2015). Among them, genogroup II genotype 4 (GII.4) is especially important in public health, because it is the leading cause of NoV-associated acute gastroenteritis in humans (Noel et al., 1999; Siebenga et al., 2007; Zheng et al., 2010). NoV is highly variable, and emergence of a novel GII.4 variant is in some cases associated with global outbreaks of gastroenteritis (Lopman et al., 2004; Vipond et al., 2004; Reuter et al., 2005; Bull et al., 2006; Gallimore et al., 2007; Motomura et al., 2008). A major shift in the pandemic strain occurs with antigenic mutations (Lindesmith et al., 2008, 2011, 2012a, 2013) and viral genome recombination at the ORF1/2 boundary region (Motomura et al., 2010; Eden et al., 2013).

The VP1 protein is the major structural protein of the mature virion, which protrudes from the virion surface and plays pivotal roles in the viral interactions with hosts. The VP1 protein is composed of two domains, protruding (P) and shell (S) (Prasad et al., 1999). The P domain is further divided into two subdomains, P1 and P2 (Prasad et al., 1999). The P2 subdomain is placed on the tip of the VP1 protein and constitutes the major antigenic site around the binding site to the putative receptor(s) for infection (Donaldson et al., 2010). This structural feature causes sequence variation (Lindesmith et al., 2008, 2011, 2012a, 2013; Bok et al., 2009; Debbink et al., 2012) and structural diversity (Chen et al., 2004, 2006; Donaldson et al., 2010), particularly in the P2 subdomain. Meanwhile, the functional importance of the P2 subdomain can cause suppression of deleterious changes and/or changes that reduce viral replication fitness. However, very little is known about evolution of the VP1 protein during viral maintenance in human populations.

To address this issue, we examined here molecular evolution of the VP1 protein of a pandemic lineage, termed GII.4_2006b, which is also known as the GII.4 Den Haag 2006b. In the autumn/winter of 2006, the national epidemiological surveillance of infectious diseases in Japan reported an unusual increase in the number of outbreaks of NoV infections (Infectious Disease Surveillance Center^1^). This augmentation was associated with the nationwide spread of a newly emerging GII.4 variant (Motomura et al., 2008), termed GII.4_2006b. The GII.4_2006b initially coexisted as a minority strain among various other NoV lineages in Japan, but starting in October of 2006 it spread extremely rapidly and remained as the major epidemic variant across Japan between 2006 and 2009 (Motomura et al., 2008, 2010). In this study, we characterized nucleotide and amino acid diversities of the VP1 proteins, using serially collected full-length 317 NoV genome and VP1 sequences from infections in Japan between 2006 and 2011. The obtained results show long-term persistent of GII.4_2006b in human populations in Japan as a dominant GII.4 subpopulation. Interestingly, both the VP1 protein and viral enzymes had remained as highly homogeneous populations, indicating strong evolutionary constraints on changes in these proteins following the onset of the outbreaks.

## Materials and Methods

### NoV Genome Sequencing

Stool specimens were collected from individuals with acute gastroenteritis at 20 regional public health institutes in Japan between May 2006 and March 2011 in compliance with the Food Sanitation Law of Japan, according to the methods for the protection of personal information (including methods for anonymization in an unlinkable fashion). The research was approved by research and ethical committee in National Institute of Infectious Diseases. Three to five stool specimens were collected at each site in each year. NoV genome sequences were obtained from the stool specimens as described previously (Motomura et al., 2008, 2010).

### Genotype Determination

Norovirus genotype was determined by construction of phylogenetic trees of viral genome sequences. Multiple sequence alignments were done as described previously (Motomura et al., 2008, 2010) using the MAFFT (Katoh et al., 2009) and alignment tools implemented in the MEGA software suite (Tamura et al., 2011). Phylogenetic trees were constructed as described previously (Motomura et al., 2008, 2010) using MEGA software (Tamura et al., 2011). The reliability of interior branches in the tree was assessed by the bootstrap method with 1,000 resamplings.

### Analysis of Diversity of Sequence Population

Mean diversity in the entire sequence population was computed with the “*Sequence Diversity*” menu in MEGA software suite (Tamura et al., 2011). The overall pairwise mean distance between the sequences was computed with the “*Distances*” menu in MEGA. As substitution models, a maximum composite likelihood and a Poisson model were used for nucleotide and amino acid sequences, respectively. Variance was estimated by the bootstrap method with 100 to 500 bootstrap replications.

### Analysis of Individual Amino Acid Variation

Amino acid variations at each position of the VP1 (1–530) were calculated as previously described with a multiple sequence alignment as described previously for other viral proteins (Naganawa et al., 2008; Oka et al., 2009; Takahata et al., 2017) on the basis of Shannon’s equation (Shannon, 1997):

$$H(i)=-\underset{x_{i}}{\Sigma }p(x_{i})\log_{2}p\left(x_{i}\right)$$

$$(x_{i}=G,A,I,V,......),$$

where *H*(*i*), *p(x*_i_), and *i* indicate the amino acid entropy score of a given position, the probability of occurrence of a given amino acid at the position, and the number of positions, respectively. An *H*(*i*) score of zero indicates absolute conservation, whereas 4.4 bits for amino acids or 2.0 bits for nucleic acids indicates complete randomness.

### Analysis of Amino Acid Substitutions by Hamming Distance

We used the Hamming distance to assess the changeability of the earliest NoV GII.4_2006b variant in Japan. In information theory, the Hamming distance between two sequences indicates the minimum number of “amino acid substitutions” required to change one sequence into the other. Because the length of amino acid sequences of the VP1 proteins of the NoV GII.4_2006b subpopulations were identical (540 amino acid residues), the Hamming distance measured in this study means the number of different amino acid residues between the earliest and subsequently emerged variants in two aligned sequences. Python was used as the programming language to compute Hamming distances. Hamming distances between the sequence in May 2006 (accession number AB447443; earliest GII.4_2006b sequence in our NoV genome dataset) and the later GII.4_2006b sequences (*n* = 249) were computed by creating a sequence that assigns mismatches and matches at corresponding positions in the two sequences, and then by counting the numbers of the mismatches.

### Nucleotide Accession Numbers

The DDBJ database accession numbers of the 317 NoV GII.4 genome sequences used in this study are provided in Supplementary Table S1 (*n* = 250, GII.4_2006b) and Supplementary Table S2 (*n* = 67, GII.4 non-2006b).

## Results

### Persistence and Diversification of NoV GII.4_2006b Genome in Japan between 2006 and 2011

**FIGURE 1:**
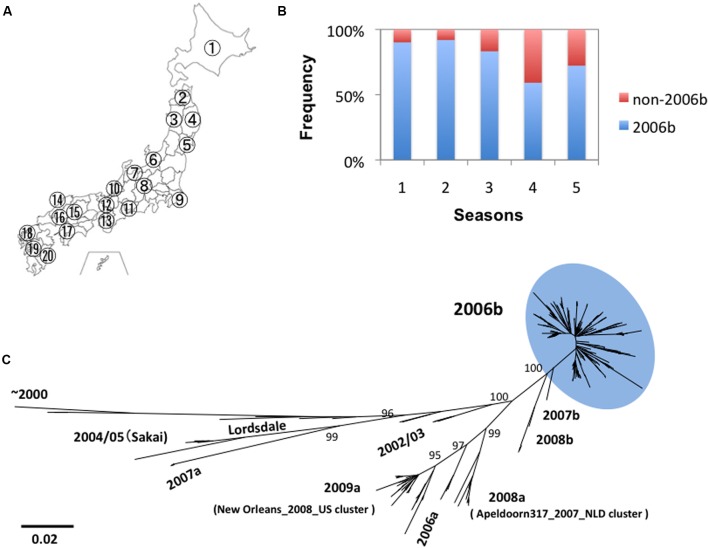
**Persistence and diversification of NoV GII.4_2006b genome in Japan between 2006 and 2011. (A)** Geographic locations of the 20 sample collection sites in Japan (see Supplementary Tables S1, S2 for the seasons and sites of collection). **(B)** Detection frequency of the GII.4_2006b variants in each period. NoV GII.4 genome sequences (*n* = 317) from stool specimens collected at the 20 sites between May 2006 and March 2011 were divided into five groups on the basis of the collection periods, i.e., 0–11 months (the season 1, *n* = 41), 12–23 months (the season 2, *n* = 75), 24–35 months (the season 3, *n* = 78), 36–47 months (the season 4, *n* = 76), and 48–58 months (the season 5, *n* = 47) after the first detection of GII.4 in May 2006 in Japan in this study. Genotypes of the GII.4 variants were determined with phylogenetic trees of the whole genome sequences as described previously (Motomura et al., 2008, 2010). The detection frequency of the GII.4_2006b genomes among the total GII.4 genomes in each collection period is shown. **(C)** Phylogenetic classification of the NoV GII.4 genome sequences used in this study. The maximum likelihood tree was constructed with the 317 GII.4 genome sequences (about 7.5 kb). The sequence cluster enclosed by a light-blue oval indicates the GII.4_2006b genomes.

We obtained 317 genome sequences of NoV GII.4 from the stool specimens collected at 20 sites in Japan between 2006 and 2011 (**Figure 1A**). Eight distinct lineages of NoV GII.4 were identified in this period. These lineages include “2004/05” related to Sakai/04-179/2005/JP cluster, “2006a” related to Yerseke 2006a cluster, “2006b” related to Den Haag 2006b cluster, “2007a,” “2007b,” “2008a” related to Apeldoorn317/2007/NL cluster, “2008b,” and “2009a” related to New Orleans 1805/2009/USA cluster (Motomura et al., 2008, 2010; Supplementary Tables S1, S2). Among the newly emerged eight GII.4 lineages, only the pandemic variant GII.4_2006b had been detected dominantly and continually throughout Japan (**Figure 1B**). The GII.4_2006b represented about 79% (*n* = 250) of the total GII.4 genomes detected during the 5 years. Phylogenetic analysis shows that the GII.4_2006b genome sequences diverged radially from a few roots (**Figure 1C**). These data suggest genetic bottlenecks followed by a rapid genome diversification of GII.4_2006b between 2006 and 2011.

### Diversity of NoV GII.4_2006b ORFs

**FIGURE 2:**
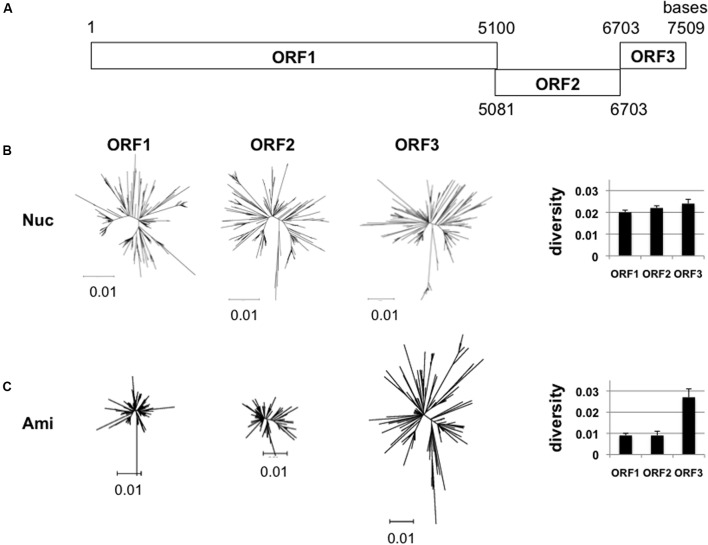
**Diversity of NoV GII.4_2006b ORFs. (A)** Schematic illustration of the locations of the three ORFs in the NoV GII.4_2006b genome. The nucleotide positions are based on the sequence of the earliest GII.4_2006b in our NoV genome dataset (Supplementary Table S1, Accession No. AB447443). **(B,C)** Neighbor-joining trees (Left) and diversity scores (Right) of the sequences of the ORF1, 2, and 3 regions in the 250 NoV GII.4_2006b genomes identified in **Figure 1C**. The analyses were done with tools included in the MEGA software suite (Tamura et al., 2011). Nucleotide sequences **(B)**. Amino acid sequences **(C)**.

The GII.4_2006b RNA genome encodes three open reading frames, ORF1, ORF2, and ORF3 (**Figure 2A**). ORF1 encodes viral enzymes and non-structural proteins. ORF2 and ORF3 encodes structural proteins, VP1 and VP2, respectively. We first examined whether the sequence diversity is different among the three ORFs by using the 250 GII.4_2006b genome sequences obtained in this study. The phylogenetic tree and mean diversity in the entire sequence population show that the nucleotide diversity was similar among the three ORFs (**Figure 2B**). In contrast, a marked difference was observed in the diversity of amino acid sequences: the ORF1 and ORF2 amino acid sequences remained significantly less diversified than that of ORF3 (**Figure 2C**). The data suggest the presence of constraints on the amino acid changes of proteins encoded by ORF1 and ORF2.

### Temporal Changes in the Sequence Diversity of NoV GII.4_2006b

**FIGURE 3:**
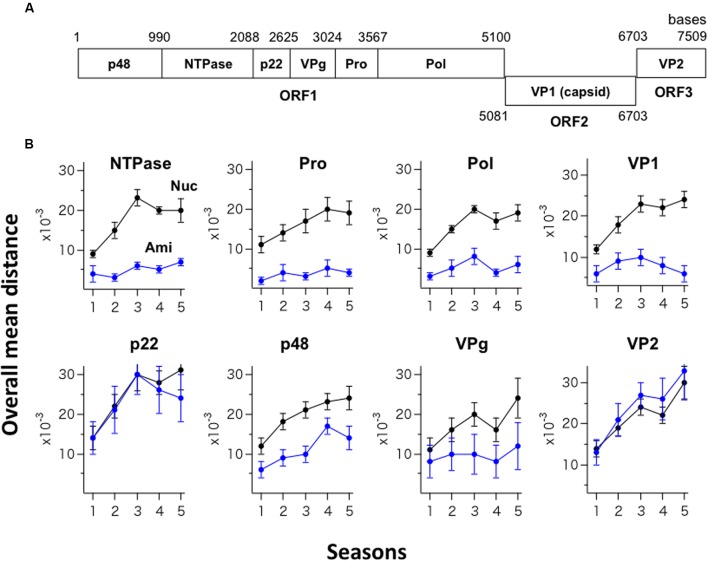
**Temporal changes in the sequence diversity of NoV GII.4_2006b. (A)** Schematic illustration of the locations of the protein-coding regions in the NoV GII.4_2006b genome. The nucleotide positions are based on the sequence of the earliest GII.4_2006b in our NoV genome dataset (Supplementary Table S1, Accession No. AB447443). **(B)** Temporal changes in dissimilarity of nucleotide and amino acid sequences in protein-coding regions of the GII.4_2006b genome. The 250 sequences of the GII.4_2006b genome were divided into five groups on the basis of the collection periods, i.e., 0–11 months (the season 1, *n* = 41), 12–23 months (the season 2, *n* = 75), 24–35 months (the season 3, *n* = 78), 36–47 months (the season 4, *n* = 76), and 48–58 months (the season 5, *n* = 47) after the first detection of GII.4_2006b in May 2006 in Japan in this study. The overall pairwise mean distance between the sequences was calculated for each season using MEGA software (Tamura et al., 2011). Black and blue circles indicate the mean distances of nucleotide (Nuc) and amino acid (Ami) sequences.

The GII.4_2006b RNA genome encodes eight viral proteins (**Figure 3A**). Shannon entropy of amino acid sequences of the 2006b ORF1 in the present genome dataset indicates that the potential sites for the internal cleavage of the ORF1 precursor protein were perfectly conserved in amino acid levels [*H*(*i*) = 0/0] for p48/NTPase (Q/G), NTPase/p22 (Q/G), and VPg/Pro (E/A), and highly conserved for p22/VPg (E/G) [*H*(*i*) = 0.03/0.08] and Pro/Pol (E/G) [*H*(*i*) = 0.03/0.03]. To assess the changeability of individual viral proteins, we examined temporal changes in the sequence diversity of the eight protein-coding regions using the 250 GII.4_2006b genomes. The genome sequences were divided into five groups based on the collected seasons, and the overall mean distance of the sequences in a season was calculated using MEGA. The nucleotide mean distance sequentially increased for the eight protein-coding regions (**Figure 3B**, Nuc), indicating a continuous increase in the dissimilarity of every gene segment in the GII.4_2006b variant population in Japan.

In contrast, the temporal change in amino acid sequence diversity was very different among the eight proteins (**Figure 3B**, Ami). Interestingly, the amino acid mean distance of the generally hypervariable VP1 protein sequences remained comparable to that of three viral enzymes (NTPase, Pro, Pol) for 5 years, with the mean distance remaining at less than 0.01 with small variances (**Figure 3B**, Upper). After the 3rd epidemic season, the VP1 amino acid distance even decreased. Meanwhile, the amino acid mean distances of the p22 and VP2 proteins sharply increased in parallel with an increase in the nucleotide distances (**Figure 3B**, p22 and VP2), suggesting the continuous diversification of these proteins in association with nucleotide diversification. The mean amino acid distance for the p48 protein increased with time yet less extensively than those for the p22 and VP2 proteins (**Figure 3B**, p48). The mean amino acid distance for the VPg protein stayed at relatively low levels with large variances (**Figure 3B**, VPg). In sum, these data suggest the presence of strong constraints on amino acid changes in the capsid protein VP1 and enzymes (NTPase, Pro, Pol) of the GII.4_2006b under the diversification of nucleotide sequences.

### Long-term Circulation of the NoV GII.4_2006b Subgroup Carrying the Identical Capsid Protein VP1

**FIGURE 4:**
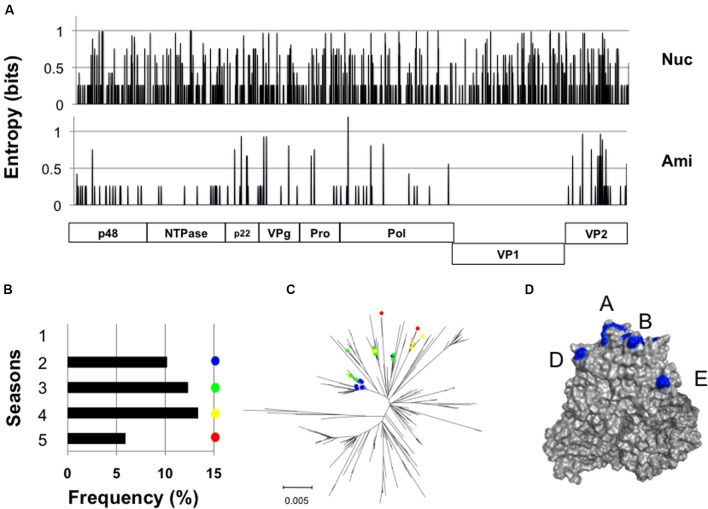
**Long-term circulation of the NoV GII.4_2006b subgroup carrying the identical capsid protein VP1. (A)** Identification of a GII.4_2006b genome subpopulation group 1 that encodes the identical VP1s in distinct genetic backbones. Nucleotide (Upper) and deduced amino acid (Lower) sequences of the group 1 genomes (*n* = 23) were aligned using MAFFT software (Katoh et al., 2009), and Shannon entropies at individual positions were calculated as described previously (Naganawa et al., 2008; Oka et al., 2009; Takahata et al., 2017). The distribution of Shannon entropy scores in the GII.4_2006b genome is shown. **(B)** Detection frequency of the group 1 genomes in five seasons between 2006 and 2011 in Japan. **(C)** Neighbor-joining tree of the GII.4_2006b VP1 nucleotide sequences (1620 nucleotides). Colored circles indicate the group 1 sequences. **(D)** P domain dimer model of the GII.4_2006b VP1 protein was constructed as described (Motomura et al., 2008, 2010). Blue residues indicate the GII.4_2006b-specific amino acid substitutions at potential epitopes in the P2 subdomain of the GII.4 VP1 (Lindesmith et al., 2012b).

We identified a GII.4_2006b subpopulation (*n* = 23) whose nucleotide sequences differed from each other, yet encoded exactly the same VP1 amino acid sequences (**Figure 4A**). The members of this population, tentatively termed group 1, were detected at distantly located 11 sample collection sites in Japan during the study period (Supplementary Table S1 and Figure S1). They emerged in the second epidemic season in 2007 and continuously circulated without no changes in the VP1 amino acid residues, representing about 6–13% of the GII.4_2006b genomes in each season (**Figure 4B**). The group 1 genomes continuously accumulated nucleotide substitutions in the VP1-coding region (**Figure 4C**), but only synonymous substitutions (**Figure 4A**). The GII.4_2006b variants at the onset of epidemics generally had 10 substitutions at the potential epitopes A, B, D, and E (Lindesmith et al., 2012a) of the VP1 P2 subdomain (P294A, T296S, T298N, A368S, D372E, M333V, R382K, S394T, D407S, and T412N), as compared with the VP1 sequence of the past epidemic variant in 2004/2005 in Japan (Sakai/04-179/2005/JP: accession number BAE98194) (**Figure 4D**). The group 1 VP1 protein had an additional substitution (S393G) on the epitope D.

### Temporal Change in Hamming Distance for the NoV GII.4_2006b Capsid Protein VP1

**FIGURE 5:**
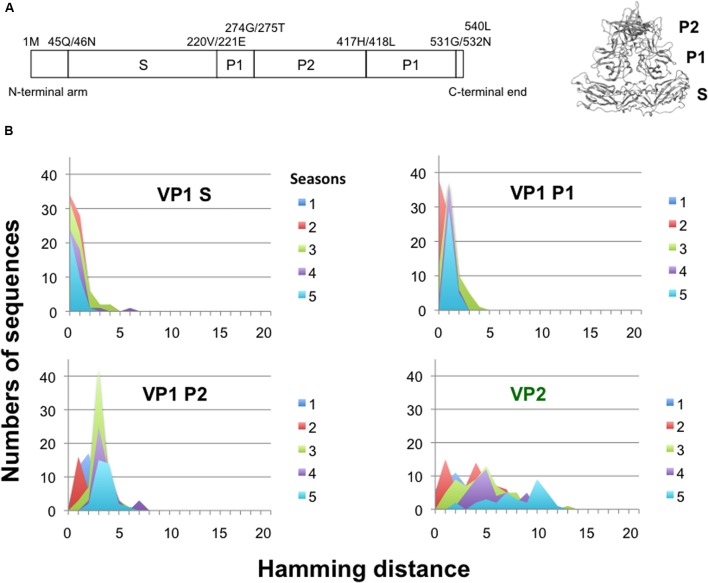
**Temporal change in Hamming distance for the NoV GII.4_2006b capsid protein VP1. (A)** Schematic illustration of the locations of the S, P1, and P2 domains in the ORF2 (Left) and the VP1 3-D model (Right) of the NoV GII.4_2006b. **(B)** Temporal changes in the Hamming distance. A GII.4_2006b genome from a stool specimen collected in May 2006 in Toyama (accession number AB447443) was the oldest in our GII.4_2006b genome dataset. The sequence was used to calculate the Hamming distance between the earliest and subsequent VP1 variants emerged between October 2006 and March 2011 in Japan. The distributions of the Hamming distances for each season are shown for the S, P1, and P2 domains of the VP1 protein. Temporal changes in the Hamming distance are also shown for the VP2 protein (Lower right).

The NoV VP1 protein has an architecture similar to that of the VP1 proteins of other single-stranded RNA viruses (Prasad et al., 1994, 1999; **Figure 5A**). The S domain is highly conserved, whereas the P2 domain is hypervariable among GII.4 variants. To assess the changeability of the P2 domain of the GII.4_2006b, we examined the temporal accumulations of amino acid substitutions in the S, P1, and P2 regions of the GII.4_2006b VP1 using the Hamming distance between the earliest and subsequent VP1 variants. As the earliest VP1 variant of the GII.4_2006b, we used a sequence from a May 2006 sample, which was collected in spring about 5 months before the onset of the nationwide epidemics of the GII.4_2006b in October of 2006 in Japan (Motomura et al., 2008).

For the S domain, the Hamming distances of the variants in given seasons were at a constant peak of 0 for 5 years (**Figure 5B**, VP1 Shell). The data suggest that the GII.4_2006b variants having amino acid substitutions in the S domain were mostly cleared during epidemics. For the P1 and P2 subdomains, the peaks of Hamming distances were fixed at 1 and 3 after the second and first epidemic seasons, respectively (**Figure 5B**, VP1 P1 and P2). The data indicate that most of the GII.4_2006b variants in the early epidemics had a few amino acid substitutions in the P domain but they could not accumulate more mutations after the second epidemic season. Thus the P domain was more variable than the S domain in the GII.4_2006b variants, as has generally been documented for other NoVs. However, the accumulation of amino acid substitutions was strictly constrained in the P domain of the GII.4_2006b variants during epidemics. In contrast, the Hamming distances of VP2, a minor structural protein in virion (Glass et al., 2000), continuously increased and showed no evidence of fixation of the peak distance during the study period (**Figure 5B**, VP2).

## Discussion

In this report, we studied molecular evolution of the NoV capsid protein of a pandemic lineage, GII.4_2006b. This NoV subpopulation predominated over other coexisting NoV GII.4 subpopulations between the 2006 and 2011 in Japan (**Figure 1**). Notably, the amino acid sequences of variable VP1 protein of the GII.4_2006b populations remained as homogeneous as that of the viral enzymes for the 5 years under an increase in nucleotide diversity (**Figures 2**, **3**). Even the GII.4_2006b population possessing the identical amino acid sequence in the VP1 protein had persisted in the study period (**Figure 4**). Even the hypervariable antigenic P2 subdomain of the VP1 protein had resisted sequential accumulations of amino acid substitutions (**Figure 5**). These results suggest the presence of strong evolutionary constraints on the VP1 protein of the NoV pandemic strain. The finding has implications for our understanding of molecular evolution, mechanisms of the periodic shifts in the pandemic NoV GII.4 strains, and control of the NoV GII.4 pandemic strain.

First, the finding has implications for understanding fitness landscape and evolution of the VP1 protein of NoV GII.4 pandemic strain. The strong constraints on changes imply that the VP1 protein and enzymes of the GII.4_2006b variants had already gained near maximal levels of fitness benefits in humans around the onset of the outbreaks and that new mutations in the VP1 protein were mostly cleared from the GII.4_2006b population, probably due to a reduction in the viral fitness for the spread in humans. In order to predominate over other coexisting GII.4 variants, the pandemic variant should have the VP1 structure that confers the best ability to evade preexisting herd immunity against NoV at that time, while also having affinity to bind to receptor(s) on human cells. Because the antigenic sites are located near the receptor-binding site, new antigenic mutations always have the risk to attenuate VP1 protein function and thereby to cause reduction in the viral replication fitness in humans. Thus, it is possible that the VP1 protein of the pandemic strain had remained conserved in human populations primarily by the necessity to maintain advantageous physical property of the VP1 protein for immune evasion and infectivity simultaneously.

Secondary, the finding has implications in the periodic shifts of the pandemic NoV GII.4 strains. Provided that the VP1 protein sequence of a given pandemic variant remained conserved following the onset of epidemics as seen in the GII.4_2006b, the human herd immunity against the VP1 protein would become increasingly more effective in association with the spread of the virus in humans. Consequently, niche for the pandemic variant in humans would be reduced, and the pandemic variant eventually be replaced by an alternative variant that has the fittest capsid structure under human herd immunity at that time. Consistently, the numbers of reported NoV infection cases in Japan had decreased annually since the late 2007, and the GII.4_2006b was replaced by a new global pandemic strain GII.4_Sydney 2012 in the 2013/2014 season, as reported in other countries (van Beek et al., 2013; Eden et al., 2014).

Finally, the finding has implications in the control of NoV pandemic strains. Although development of vaccines and antiviral agents are of special importance to reduce damages from the NoV infections, structural variations in the viral proteins can be problematic. In this regard, the present study suggests the presence of strong constraints on changes in capsid protein and enzymes of a NoV GII.4 pandemic variant on the course of 5-year persistence across Japan. The finding provides a rationale for developing vaccines and antiviral agents against a pandemic strain. A basic premise of the control is that the sequences of the VP1 protein and viral enzymes of a given pandemic variant remain highly homogeneous after the onset of pandemic. Therefore, it is important to further accumulate information on the evolution of newly emerged pandemic strains to clarify whether present observations of the amino acid conservation in the VP1 and viral enzymes can be extended to other GII.4 pandemic variants. In parallel, it would be important to study genetic diversity of NoV in nature in order to develop systems to predict a new pandemic variant in advance.

## Author Contributions

HS conceived the study. MY prepared the computing environment for information science. KK, NT, MN, and TT organized collection of stool specimen. TO, HN, and KM performed sequencing. HN and HS performed variation analysis. HS prepared the manuscript. All authors read and approved the final manuscript.

## Conflict of Interest Statement

The authors declare that the research was conducted in the absence of any commercial or financial relationships that could be construed as a potential conflict of interest.

## Footnotes

1 http://idsc.nih.go.jp/iasr/prompt/graph-ke.html
