# Supplementary figures and images for: Evolutionary Constraints on the Norovirus Pandemic Variant GII.4_2006b over the Five-Year Persistence in Japan
Source: Front Microbiol. 2017 Mar 13;8:410. doi: 10.3389/fmicb.2017.00410 (PMC5346551; doi:10.3389/fmicb.2017.00410)

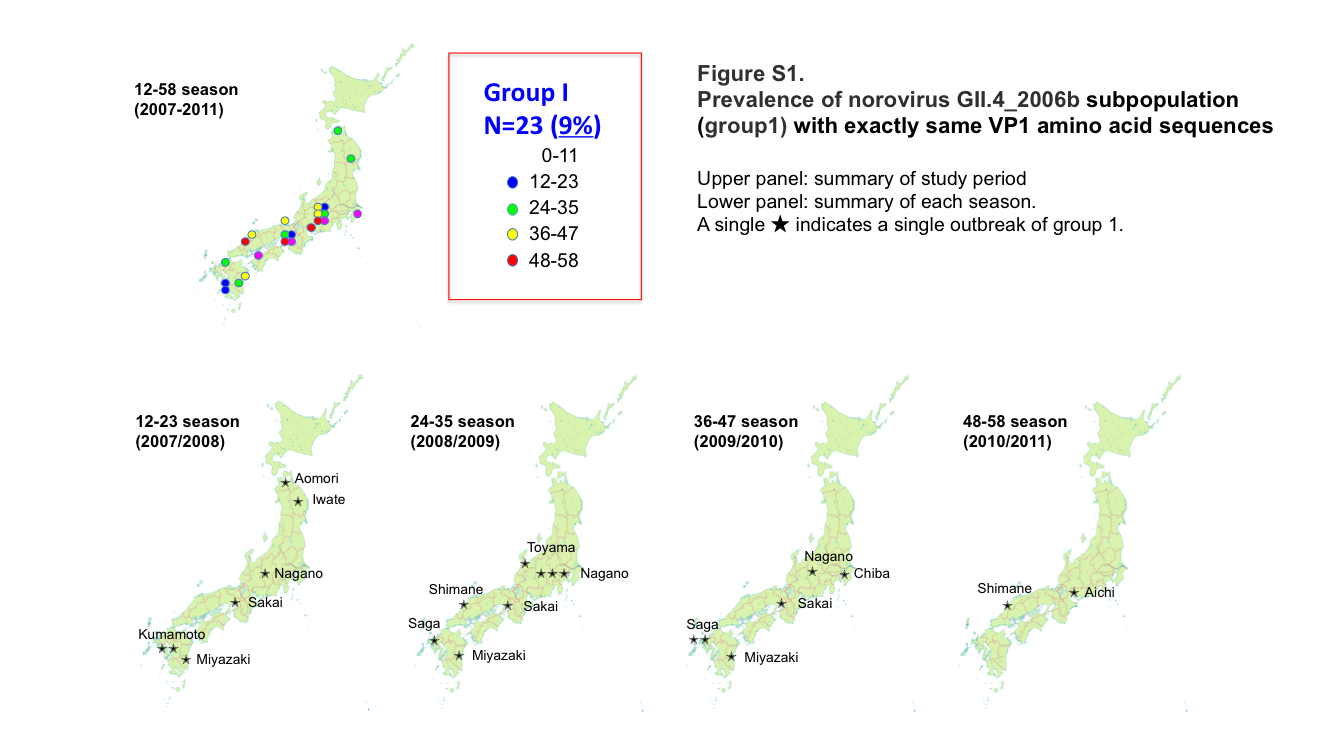

Supplement: Supplementary file 3 [file Image_1.TIF]
